# On Referring Unread Papers

**Published:** 1859-07

**Authors:** 


The custom in our medical societies, of referring unread papers to the Committee on Publication, we think, a wrong and injurious one.

In the first place, we should imagine no one would be willing to prepare a paper, giving it all the care, attention and research necessary to do it properly, and justice to himself, would be willing to donate his services for such a purpose with no other equivalent than seeing his name and paper in print.

One of the principal objects for which societies are formed, is for the interchange of opinion, and to elicit thoughts and ideas, and what serves as so fruitful a basis or text, as the several papers there presented; therefore, we say, a writer should be unwilling to allow his paper to pass undiscussed, that he, in common with the rest, might receive his reciprocal share of profit.

In the next place, courtesy demands that the society, unless for very good and sufficient reasons, should listen to the results of the labors of its members.

Many of the papers are prepared with great care, and are abundantly worthy of the attention of the Society, and to be placed among its recorded labors; others are not.

The position of the Committee of Publication is such, that very little option is granted them, and a reference is almost, or entirely equivalent to an order for publication. The Society should hear, and then have independence enough to decide upon the worth or inferiority of papers presented.

The amount of business, as yet, of the Illinois State Medical Society, is no excuse, and the absence of the writer can easily be remedied in a substitute.

The difficulty is yet comparatively small, and we only allude to the subject that it may not be allowed thoughtlessly to grow into a serious evil, as it has in many States. R.

